# Supplementary material for: The Impact of Phenotypic Heterogeneity on Chemotactic Self-Organisation
Source: Bull Math Biol. 2022 Nov 1;84(12):143. doi: 10.1007/s11538-022-01099-z (PMC9626439; doi:10.1007/s11538-022-01099-z)
Supplement: Supplementary file 5 — (pdf 80 KB) [file 11538_2022_1099_MOESM5_ESM.pdf]

Supplementary Material for: 'The impact of phenotypic heterogeneity on chemotactic self-organisation'

Fiona R Macfarlane, Tommaso Lorenzi and Kevin J Painter

Supplementary material 1 (SM1)

Video to support Figure 4 in the main text. Video displays the 1D spatial distribution of the cell densities n_0 and n_1 over time for the simulations corresponding to each form of the phenotypic switching functions given in Table 1 in the main text. The parameter setting used is $\mu = 1$, $q = 30$ and χ equal to 15 (Case B_1), 5 (Case B_2), 75 (Case C_1), 10 (Case C_2). All other parameters, the initial condition and numerical set-up are provided in Appendix D.2 in the main text.

Supplementary material 2 (SM2)

Video to support Figure 5 in the main text. Video displays the 2D spatial distribution of the cell densities n_0 and n_1 over time for the simulations corresponding to each form of the phenotypic switching functions given in Table 1 in the main text. The parameter setting used is $\mu = 1$, $q = 1$ and χ equal to 10 (Case A), 15 (Case B_1), 5 (Case B_2), 10 (Case C_1), 10 (Case C_2). All other parameters, the initial condition and numerical set-up are provided in Appendix D.2 in the main text.

Supplementary material 3 (SM3)

Video to support Figure 6 in the main text. Video displays the 2D spatial distribution of the cell densities n_0 and n_1 over time for the simulations corresponding to three forms of the phenotypic switching functions given in Table 1 in the main text. The parameter setting used is $\mu = 1$, $q = 30$ and χ equal to 15 (Case B_1), 5 (Case B_2), 75 (Case C_1). All other parameters, the initial condition and numerical set-up are provided in Appendix D.2 in the main text.

Supplementary material 4 (SM4)

Video to support Figure 7 in the main text. Video displays the 2D spatial distribution of the cell density n_1 over time for the simulations corresponding to different parameter settings. In all cases, the functions μ_{01} and μ_{10} are taken to be those of Case B_2 in Table 1. For each panel, the parameter setting investigated is highlighted by the panel titles. All other parameters, the initial condition and numerical set-up are provided in Appendix D.2 in the main text.
